# Spectrum of Beta Globin Gene Mutations in Egyptian Children with β-Thalassemia

**DOI:** 10.4084/MJHID.2014.071

**Published:** 2014-11-01

**Authors:** MR El-Shanshory, AA Hagag, SS Shebl, IM Badria, AH Abd Elhameed, ES Abd El-Bar, Y Al-Tonbary, A Mansour, H Hassab, M Hamdy, M Alfy, L Sherief, E Sharaf

**Affiliations:** 1 Pediatric Department, Tanta University, Egypt; 2 Clinical Pathology Department, Tanta University, Egypt; 3 Pediatric Departments of Mansoura University; 4 Pediatric Departments of Alexandria University; 5 Pediatric Departments of Cairo University; 6 Pediatric Departments of Ain Shams University; 7 Pediatric Departments of Zagazeg University; 8 Pediatric Departments of Sohag University

## Abstract

**Background:**

The molecular defects resulting in a β-thalassemia phenotype, in the Egyptian population, show a clear heterogenic mutations pattern. PCR-based techniques, including direct DNA sequencing are effective on the molecular detection and characterization of these mutations. The molecular characterization of β-thalassemia is necessary for carrier screening, genetic counseling, and to offer prenatal diagnosis.

**The aim of the work:**

was to evaluate the different β-globin gene mutations in two hundred β-thalassemic Egyptian children.

**Subjects and Methods:**

This study was carried out on two hundred β-thalassemic Egyptian children covering most Egyptian Governorates including 158 (79%) children with thalassemia major (TM) and 42 (21%) children with thalassemia intermedia(TI). All patients were subjected to meticulous history taking, clinical examination, complete blood count, hemoglobin electrophoresis, serum ferritin and direct fluorescent DNA sequencing of the β-globin gene to detect the frequency of different mutations.

**Results:**

The most common mutations among patients were IVS I-110(G>A) 48%, IVS I-6(T>C) 40%, IVS I-1(G>A) 24%, IVS I-5(G>C)10%, IVS II-848 (C>A) 9%, IVS II-745(C>G) 8%, IVS II-1(G>A) 7%, codon “Cd”39(C> T) 4%, −87(C>G) 3% and the rare mutations were: Cd37 (G>A), Cd8 (−AA), Cd29(−G), Cd5 (−CT), Cd6(−A), Cd8/9(+G), Cd 106/107(+G), Cd27(C>T), IVS II-16(G> C), Cd 28 (−C), Cap+1(A>C), −88(C>A), all of these rare mutations were present in 1%. There was a considerable variation in phenotypic severity among patients resulting from the interaction of different β^∘^ and β+mutations. Furthermore, no genotype-phenotype association was found both among the cases with thalassemia major and the cases with thalassemia intermedia.

**Conclusion:**

Direct DNA sequencing provides insights for the frequency of different mutations in patients with β-thalassemia including rare and/or unknown ones. The most common mutations in Egyptian children with beta thalassemia were IVS I-110(G>A) 48%, IVS I-6(T>C) 40%, IVS I-1(G>A)24%, IVS I-5(G>C)10%, IVS II-848 (C>A) 9%, IVS II-745(C>G) 8%, IVS II-1(G>A) 7%.

## Introduction

Thalassemia syndrome is the most common single gene disorder.^1^ It is an autosomal recessive hereditary anemia due to mutations that reduce (β+) or abolish (β ^∘^) synthesis of β-globin chains of hemoglobin tetramer, which is made of two alpha and two beta globin chains (α2 & β2) required for HbA formation.^2^ The disease is very heterogeneous at the molecular level, with more than 300 different molecular defects defined to date.^3^

As in many Mediterranean countries, β-thalassemia is a major public health problem in Egypt. The position of Egypt in the center of the Middle East, contiguous with the Mediterranean countries, has facilitated genetic admixture of Egyptians with several populations of diverse geographic and ethnic origins.^4^

It has been estimated that 1000 children out of 1.5 million live births are born annually with thalassemia major.^5^ In multicenter studies, the carrier rate in Egypt has been reported to be in the range of 9%–10%.^4^

Treatment of β-thalassemia, albeit more and more available, remains a significant drain on the country’s resources. Regular blood transfusions in combination with iron chelation have remarkably increased the lifespan of patients with β-thalassemia.^6^

However, iron-related complications, including life-threatening ones such as heart disease, are still common. A prevention program would be useful to overcome these problems, but it requires a preliminary knowledge of the most common β-globin mutations among the population.^7^

DNA sequencing as availability of this method and standardization of this technique in the country can help in choosing the best strategy for molecular diagnosis with the possibility to detect rare mutations in the area.^8^

The present work aimed to evaluate the different β-globin gene mutations in two hundred of Egyptian children with β-thalassemia by direct DNA sequencing to be taken in consideration of prevention program of β-thalassemia.

## Subjects and methods

This study was conducted on 200 cases of children with β-thalassemia including 158 children with thalassemia major and 42 children with thalassemia intermedia. An informed consent was obtained from all parents of children before enrollment in the study. The study was approved by the Ethical Committee of Tanta University.

These children came from most of Egyptian Governorate with a random selection from thousand cases (Alexandria, Cairo, Al-Gharbiyah, Al Manofia, Kafr El Sheikh, Sohag, Al-Fayoum, Al Kaluobia, Port Said, Al-Dakhlia, Domiat, Al Jizaz, and Al-Beheira). The rest traces from other Governments.

All patients were subjected to meticulous history taking with reference to positive consanguinity and clinical evaluation of all body systems. All affected patients were clinically classified into thalassemia major or intermedia with consideration to: age of disease onset, age of first transfusion, frequency of blood transfusion, hemoglobin level, hepatosplenomegaly, facial and growth affection.^8^

Routine hematological investigations e.g.: complete blood count using ERMA PCE-210 N cell counter, reticulocyte count, Hb electrophoresis using cellulose acetate in a tris EDTA borate buffer at PH 8.4 (Helena Laboratories, Beaumont, TX, USA), serum ferritin levels using Monobind Inc ELISA Microwells kit (lake Forest, CA 92630, USA).

Children with beta thalassemia major and intermedia were studied with DNA sequencing: DNA extraction and purification was performed from whole blood collected in EDTA-containing tubes, by using a QIA amp DNA blood mini kit (Qiagen, Hilden, Germany CA. No. 51104), according to the manufacturer’s instruction.

The PCR amplification products of each sample were applied to gel electrophoresis (2% agarose gel stained with ethidium bromide) and visualized under UV illumination (Biometra Germany). The samples were detected as a clear, sharp, distinct band at the specific molecular weight (550 bp, for Hemoglobin subunit beta-1 (HBB1), 650 bp for Hemoglobin subunit beta-2) (HBB2). The positive PCR products were then purified by PCR purification columns, using QIA Quick^R^ PCR Purification kit (Qiagen, Hilden, Germany cat. No. 28104). Then subjected to cycle sequencing PCR using fluorescent dyes (Applied Biosystems, Foster City, CA, USA).

Following the cycle sequencing PCR, the samples were then purified to remove low molecular weight components like nucleotides and buffer salts, using CENTRI-SEP columns (cat. No. CS-901). The cycle Sequence products were then analyzed with an automated sequencer (ABI PRISM™ 310 Genetic Analyzer). Finally: Interpretations of the results via SeqScape software version 2.7 Applied Biosystem.^9^ The Primer sequences are not available from the manufacturer”.

### Statistical Analysis

Data were analyzed using SPSS version 20. Data were expressed as mean ± standard deviation for quantitative variables, number and percentage for qualitative ones with the use of Chi-square, ANOVA tests. P value < 0.05 was considered to be statistically significant.

## Results

There were no significant differences between patients with thalassemia major and thalassemia intermedia regarding age, sex, family history of thalassemia, consanguinity (fifty-five percent of thalassemic patients had positive consanguinity, and 40% had a positive family history of thalassemia (presence of one brother or sister suffering from thalassemia), weight, height and body mass index (BMI).

Pallor and jaundice were the most common presenting symptoms while hepatomegaly and splenomegaly were the most common presenting signs in patient’s group.

The age of 1^st^ transfusion in studied patients ranged from 4–72 months, with a mean age of first transfusion of 16.52 ± 5.96 months, and interval of transfusion ranged from 2–24 weeks with mean interval of 4.09 ± 2.29 weeks. There were significantly lower red blood cells (RBCs), hemoglobin (Hb), and significantly higher reticulocytes, platelets and white blood cells (WBCs) in patients with thalassemia major compared with patients with thalassemia intermedia with no significant differences between patients with thalassemia major and thalassemia intermedia as regard mean corpuscular volume (MCV) and mean corpuscular hemoglobin (MCH) (Tables 2,3).

There was significantly lower total iron binding capacity, and significantly higher serum ferritin and serum iron, in patients with thalassemia major compared with patients with thalassemia intermedia (serum ferritin was 2857 ± 146 ng/dl in thalassemia major versus 910 ± 123 ng/dl in thalassemia intermedia with p value < 0.001).

Globin mutations are presented in table 4 including common and rare mutations as follow:

### Common mutations

The most common mutations among patients were IVS I-110(G>A), which was present in 96 cases out of two hundred (48%), homozygous pattern was present in 40 cases of them; compound heterozygous with other mutations was present in 56 cases; IVS I-6(T>C), which was present in 80 cases (40%), 10 of them were homozygous and 70 were compound heterozygous; IVS I-1(G>A), which was present in 48 cases (24%), 8 of them were homozygous, and 40 were compound heterozygous; IVS I-5(G>C), which was present in 20 cases (10%), 2 of them was homozygous, and 18 were compound heterozygous; IVS II-848(C>A), which was present in 18 cases (9%), 2 of them was homozygous, and 16 were compound heterozygous; IVS II-745(C>G), which was present in 16 cases (8%), 4 of them were homozygous, and 12 were compound heterozygous; IVS II- 1(G>A), that was present in 14 cases (7%), 2 of them was homozygous, and 12 were compound heterozygous; Cd39(C>T), which was present in 8 cases (4%), 2 of them was homozygous, and 6 were compound heterozygous; −87(C>G), which was present in 6 cases (3%), all of them were compound heterozygous.

### Rare mutations

Cd37 (G>A), Codon 8 (−AA), Cd29(−G), Codon5 (−CT), cd6(−A), Cd8/9(+G), Cd 106/107(+G), Cd27(C>T), IVS II-16(G> C), Codon 28 (−C), Cap+1(A>C), −88(C>A) all of these rare mutations were present in 1% all of them were compound heterozygous, except for Cd37 (G>A), and IVS II-16(G> C) were homozygous.

## Discussion

β-Thalassemia is the most common genetically inherited hemoglobin disorder in Egypt.^11^ The molecular defects resulting in a β-thalassemia phenotype, in the Egyptian population show a clear heterogenic pattern. Many studies have embarked on the molecular detection and characterization of these mutations, using a wide array of the available techniques with successful detection of both known and unknown mutations. PCR-based techniques, including direct DNA sequencing are effective with some limitations about the time, effort and high cost to reach a final diagnosis.^12^

The aim of this work was to evaluate the different β-globin gene mutations in two hundred β-thalassemic Egyptian children.

In the present study, the most common mutations among patients were IVSI-110(G>A) which were present in 96 cases out of two hundred (48%), and IVSI-6(T>C) was present in 80 cases (40%), then IVSI-1(G>A) in 48 cases (24%), IVSI-5(G>C) in 20 cases (10%), IVSII-848(C> A) in 18 cases (9%), IVSII-745(C> G) in 16 cases (8%), IVSII-1(G>A) in 14 cases (7%), Cd39(C> T) in 8 cases (4%), −87(C> G) in 6 cases (3%).

These results were in agreement with Hussein et al., 2007,^13^ who found 12 different mutations in patients from Suez Canal region; the most frequent mutations were IVSI-110 (G→A) (31.4%), IVSI-1(G→A)(17.6), IVSI-6(T→C)(17.6%), −87(C>G)(7.8%), IVSII-1(G>A)(5.9%), IVSII-745(C> G)(5.9%).

This study was in accordance with Kaddah et al., 2009,^14^ who reported that the most common seven genetic mutations of the β thalassemia evaluated in Egyptian studies were IVSI-6, IVSI-110, IVSII-1, IVSII-745, IVSI-1, −87 and codon 39. Also Settin et al., 2006^15^ stated that three abundant mutations were found accounting for a total 71.25% of all mutations; these 3 mutations were IVS I-110 (G→A), IVS I-6 (T→C) and IVS I-1 (G→A) representing 37.5%,17.5% and 16.25% respectively.

Jiffri et al., 2010^16^ in another specified study of upper Egypt agreed with our study finding that the most frequent mutation was IVS-I-110 (G→A) (57%). The IVS-I-110, IVS-I-6 (T→C) and IVS-I-1 (G→A) mutations accounted for 87% of mutations in the β-thalassemia.

Consistent with this study Omar et al., 2005,^17^ in Alexandria, reported the most common mutations are IVSI-110(62%) followed by IVSI-6(7%) and IVSI-1 (4%), other mutations IVSII-1 & Cd-39 are not found in any of the studied patients.

On the other hand El-Gawhary et al., 2007,^18^ reported that IVSI-6 is more frequent than IVSI-110, but their study covered Fayoum in Upper Egypt, Cairo, Alexandria and Tanta in Lower Egypt and the Nile Delta. The proportion of IVS-I-6 (T→C) was 36.3% and of IVSI-110 (G→A) 25.8%.

Rare mutations in our study: Cd37(G>A), Cd8(−AA), Cd29(−G), Cd5(−CT), Cd6(−A), Cd8/9(+G), Cd106/107(+G), Cd27(C>T), IVSII-16(G> C), Cd28(−C), Cap+1(A>C), −88(C>A) all of these rare mutations were present in 1%.

There was a considerable variation in phenotypic severity among patients resulting from interaction of different β ^∘^ and β+mutations, 158 (79%) cases were thalassemia major (TM) and 42 (21%) were thalassemia intermedia (TI). This result was in agreement with Nadkarni et al., 2007,^10^ and Omar et al., 2005.^17^

## Conclusion

Direct DNA sequencing provides insights for the frequency of different mutations in patients with β-thalassemia including rare and /or unknown ones. The most common mutation in Egyptian children with beta thalassemia were IVS I-110(G>A) 48%, IVS I-6(T>C) 40%, IVS I-1(G>A) 24%, IVS I-5(G>C) 10%, IVS II-848 (C>A) 9%, IVS II-745(C>G) 8%, IVS II-1(G>A) 7%.

## Figures and Tables

**Figure 1:**
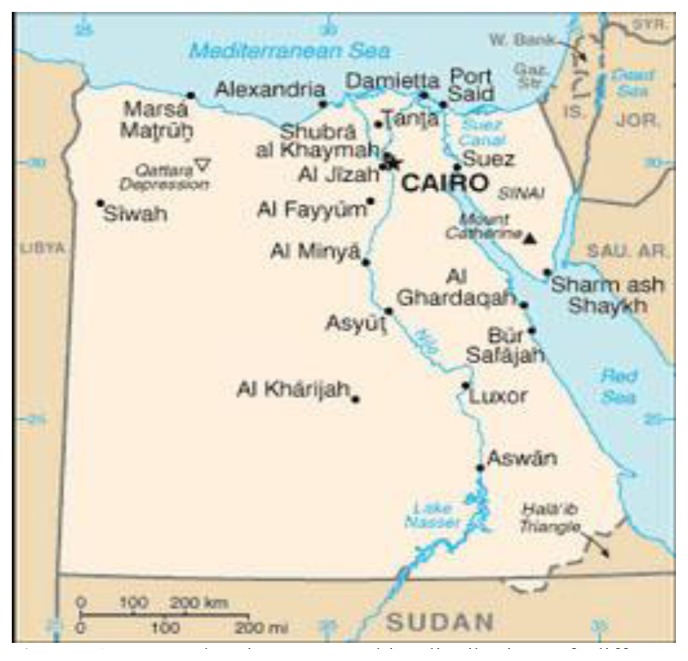
Map showing geographic distribution of different governorates in Egypt

**Figure 2:**
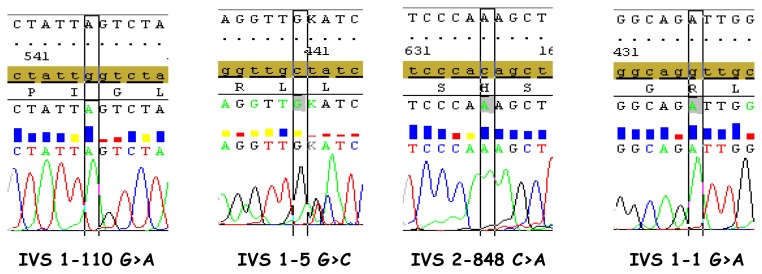
Examples of SeqScape elctropherograms.

**Table 1:** Geographical distribution of the cases covering Egyptian governorate.

**Alexandria**	**20**
**Al Fayoum**	**11**
**Cairo**	**9**
**El Beheira**	**22**
**Sohag**	**10**
**Asiout**	**10**
**Al Gharbiyah**	**20**
**Giza**	**13**
**Domiatt**	**12**
**Dakahlia**	**9**
**Port Said**	**11**
**Kaluobia**	**9**
**Al Monofia**	**16**
**Kafr El sheikh**	**18**
**Al Sharkia**	**10**
**Total**	**200**

**Table 2:** Comparison of serum ferritin and pre-transfusion complete blood count in patients with Thalassemia major and Thalassemia intermedia

	TM Patients (no=158)	TI Patients (no=42)	X^2^	P-value

**RBCs (million cell/mm^3^)**				
**Range**	3.3–4	3.5–4.4		
**Mean ± SD**	3.20 ± 0.69	4.15 ± 0. 25	10.78	<0.001*

**Hb (g/dl)**				
**Range**	4.9–9	10–11.2		
**Mean ± SD**	7.61± 1.27	10.79±−0.59	16.286	<0.001*

**MCV (fl)**				
**Range**	52.6–75	60.8–79.6		
**Mean ± SD**	63.18 ± 7.32	68.64±1.97	1.976	0.058

**MCH (pg)**				
**Range**	15.1–22	22–28.8		
**Mean ± SD**	19.57±2.16	23.33±1.06	1.474	0.152

**WBCs (cells/mm^3^**)				
**Range**	4.5–26.5	4.5–9.8		
**Mean ± SD**	13.81±5.49	6.79±1.65	6.7	<0.001*

**Platelets/mm^3^**				
**Range**	264.4–699.6	280–430.5		
**Mean ± SD**	482±217.6	335±74.5	−3.5	<0.001*

**Reticulocytes**				
**Range**	3.5–8.6	2.5–4.5		
**Mean ± SD**	4.86±1.46	3.6±0.04	1.35	0.62

**Serum ferritin**				
**Range**	2670–2990	800–1030		
**Mean ± SD**	2857 ± 146 ng/ml	910 ± 123 ng/ml		< 0.001

TM = thalassemia major. TI= thalassemia intermedia.

* Significant (P<0.05).

**Table 3:** Clinical presentation of the studied cases

Mutation	TM (no=158)	TI (no=42)

**Age / year**		
Mean ±SD	1.42± 0.69	6.22± 2.38
Range	1–17	6–17

**Age at diagnosis/month**		
Mean ±SD	15.36 ±5.36	36.42 ± 4.13
Range	3–14	35–72

**Age of 1^st^** **transfusion/month**		
Mean ±SD	16.52 ± 5.96	46.52 ± 5.96
Range	4–72	36–72

**Interval of transfusion/week**		Non transfusion dependent
Mean ±SD	4.05± 2.29	
Range	2–24	
Every 2 weeks	8%	
Every 3 weeks	25%	
Every 4 weeks	52 %	
Every 5 weeks	7%	
Every 6–8 Weeks	7 %	
Every 24 weeks	1%	

**Pallor**	93 %	26%

**Jaundice**	41%	9%

**Hepatomegaly**	78%	12%

**Splenomegaly**	25%	10%

**Splenectomy**	22%	None

TM = thalassemia major. TI= thalassemia intermedia

Severity index used to classify patients into thalassemia major and intermedia includes: Age at presentation, age of first transfusion, degree of liver enlargement, degree of spleen enlargement, baseline Hb (Pre transfusion or at the time of diagnosis). Points assigned for each patient were added to determine the SI:>8= Thalassemia major and ≤ 8= Thalassemia intermedia.**10**

**Table 4:** Different Globin mutations among the studied cases

Mutation	TM (no=158)	TI (no=42)

**IVS I-110 (G>A) (no=96 cases)**	**78**	**18**

Homozygous pattern	34	6
Compound heterozygous with other mutations	44	12

**IVS I-6(T>C) (no=80 cases)**	**60**	**20**

Homozygous pattern	6	4
Compound heterozygous with other mutation**s**	54	16

**IVS I-1(G>A) (no=48 cases)**	**36**	**12**

Homozygous pattern	6	2
Compound heterozygous with other mutations	34	6

**IVS I-5(G>C) (no=20 cases)**	**16**	**4**

Homozygous pattern	2	-
Compound heterozygous with other mutations	16	2

**IVS II-848(C>A) (no=18 cases)**	**16**	**2**

Homozygous pattern	2	**-**
Compound heterozygous with other mutations	14	**2**

**IVS II-745(C>G) (no=16 cases)**	**16**	**-**

Homozygous pattern	4	-
Compound heterozygous with other mutations	12	-

**IVS II- 1(G>A) (no=14 cases)**	**10**	**4**

Homozygous pattern	2	**-**
Compound heterozygous with other mutations	8	**4**

TM = thalassemia major. TI= thalassemia intermedia

**Table 5:** Percentage of IVSI-110 (G>A) mutation in different areas of Egypt

	Our study	Suez canal	Mansoura	Alexandria	Cairo
**Number of cases**	200	35 families	25	50	95
**Method**	DNA sequencing	PCR Direct sequencing	PCR ASO hybridization	PCR ASO hybridization Direct sequencing	PCR ARMS
**Percentage of IVSI-110 (G>A) mutation**	48%	31.4	27.1	31.8	25.8
**References**		**Hussein et al 2007** **^(^**13**^)^**	**Novelleto et al 1990** **^(^**19**^)^**	**Omar et al 2005** **^(^**17**^)^**	**Elgawhary et al 2007** **^(^**18**^)^**
